# Genome-wide identification of *CCO* gene family in cucumber (*Cucumis sativus*) and its comparative analysis with *A. thaliana*

**DOI:** 10.1186/s12870-023-04647-4

**Published:** 2023-12-11

**Authors:** Jannat Akram, Riffat Siddique, Muhammad Shafiq, Bushra Tabassum, Muhammad Tariq Manzoor, Muhammad Arshad Javed, Samia Anwar, Bader Un Nisa, Muhammad Hamzah Saleem, Bilal Javed, Tabarak Malik, Abd El-Zaher M. A. Mustafa, Baber Ali

**Affiliations:** 1 Department of Botany, Lahore College for Women University, Lahore, 54000 Pakistan; 2 Department of Horticulture, Faculty of Agricultural Sciences, University of the Punjab, Lahore, 54590 Pakistan; 3 School of Biological Sciences, University of the Punjab, Lahore, 54590 Pakistan; 4 Department of Plant Breeding and Genetics, Faculty of Agricultural Sciences, University of the Punjab, Lahore, 54590 Pakistan; 5 College of Plant Science and Technology, Huazhong Agricultural University, Wuhan, 430070 China; 6 Department of Biomedical Sciences, Institute of Health, Jimma University, 378 Jimma, Ethiopia; 7 Department of Botany and Microbiology, College of Science, King Saud University, 11451 Riyadh, Saudi Arabia; 8 Department of Plant Sciences, Quaid-I-Azam University, Islamabad, 45320 Pakistan

**Keywords:** *CCO* gene family, *C. sativus*, Chitosan oligosaccharides, Phloem content

## Abstract

Carotenoid cleavage oxygenase (CCO) is an enzyme capable of converting carotenoids into volatile, aromatic compounds and it plays an important role in the production of two significant plant hormones, i.e., abscisic acid (ABA) and strigolactone (SL). The cucumber plant genome has not been mined for genomewide identification of the CCO gene family. In the present study, we conducted a comprehensive genome-wide analysis to identify and thoroughly examine the CCO gene family within the genomic sequence of Cucumis sativus L. A Total of 10 CCO genes were identified and mostly localized in the cytoplasm and chloroplast. The CCO gene is divided into seven subfamilies i.e. 3 NCED, 3 CCD, and 1 CCD-like (CCDL) subfamily according to phylogenetic analysis. Cis-regulatory elements (CREs) analysis revealed the elements associated with growth and development as well as reactions to phytohormonal, biotic, and abiotic stress conditions. CCOs were involved in a variety of physiological and metabolic processes, according to Gene Ontology annotation. Additionally, 10 CCO genes were regulated by 84 miRNA. The CsCCO genes had substantial purifying selection acting upon them, according to the synteny block. In addition, RNAseq analysis indicated that CsCCO genes were expressed in response to phloem transportation and treatment of chitosan oligosaccharides. CsCCD7 and CsNCED2 showed the highest gene expression in response to the exogenous application of chitosan oligosaccharides to improve cold stress in cucumbers. We also found that these genes CsCCD4a and CsCCDL-a showed the highest expression in different plant organs with respect to phloem content. The cucumber CCO gene family was the subject of the first genome-wide report in this study, which may help us better understand cucumber CCO proteins and lay the groundwork for the gene family's future cloning and functional investigations.

**Supplementary Information:**

The online version contains supplementary material available at 10.1186/s12870-023-04647-4.

## Introduction

Carotenoids, which are naturally occurring isoprenoid compounds, exhibit widespread distribution across various organisms including bacteria, prokaryotes and plants [1, 2]. Since their discovery in the nineteenth century, more than 700 distinct carotenoids have been identified and characterized [3]. In the realm of plants, carotenoids have been found to fulfill diverse roles in essential biological processes [4]. They serve as photosynthetic pigments and general antioxidants, facilitating light capture and prevent photo oxidation [5]. Throughout the process of carotenoid cleavage into various apocarotenoid products, they assume various functions, including the regulation of plant growth and development and also show response to biotic and abiotic stresses in plants [6].

Plants rely heavily on the Carotenoid Cleavage Oxygenase (*CCO*) family because they are essential for controlling carotenoid metabolism. The cleavage of carotenoids by *CCO* enzymes results in the creation of several apocarotenoids, which are important signaling molecules and building blocks for the synthesis of hormones and fragrance chemicals [7]. Numerous physiological processes, such as plant development, stress responses, and the regulation of defense systems against diverse environmental challenges, are facilitated by the diverse roles of *CCOs*. Furthermore, the capacity of *CCOs* to produce bioactive substances like retinoids and abscisic acid highlights the importance of these entities in regulating plant growth and adaptive responses. The complex role that the *CCO* family plays in carotenoid metabolism places it at the forefront of influencing the many facets of plant biology and adaptability [8].

Carotenoid cleavage oxygenases (*CCOs*), which are classified as non-heme iron oxygenases cause the oxidative alteration of carotenoids that yields apocarotenoids. Plants use apocarotenoids as pigments, phytohormones and defensive chemicals [9]. Enzymes, which are also known as carotenoid cleavage dioxygenases (*CCDs*) in some scientific literature, need deoxygenate to begin the oxidative cleavage process [10]. In this study, we have opted to refer to them as *CCOs* for clarity, to distinguish them from the *CCD* subfamily.

The *CCO* gene family has been extensively investigated and characterize in numerous plant species, including *Oryza sativa* [11], *Arabidopsis thaliana* [12], *Solanum Lycopersicum* [13] and *Capsicum* [14]. Based on how well their substrate contributes to an epoxy structure, the *CCOs* can be further separated into nine-cis-epoxide carotenoid dioxygenase (*NCED*) and carotenoid cleavage dioxygenase (*CCDs*) [11].

Previous studies have shown the various roles played by genes from the *CCD* subfamily in plant physiology and development. Aside from these functions, *CCD* is also involved in photosynthesis, reactions to biotic and abiotic stressors, the production of apocarotenoids, including aromatic volatiles and strigolactones (SLs) and other processes [15]. In the case of tomatoes, the *LeCCD1* gene is involve in the production of essential flavor volatiles [16]. Similar to this, *PhCCD1* in petunias is linked to the production of β-ionone, an essential component of the smell present in many plant species Additionally, *CCD1* enzymes affect how plants react to stress [17]. During leaf senescence, for instance, *CCD1* expression increases, suggesting that *CCD1* enzymes may be involved in the catalysis of carotenoids and apocarotenoids [18].

Abscisic acid (ABA) is synthesized by the *NCED* subfamily of genes, which has been thoroughly characterized for this interaction [19]. ABA is well-known for its fundamental roles in plant developmental processes and stress signaling [20]. 9-cis-epoxy carotenoids cleave to create the precursor of ABA known as C15-xanthoxin [21]. Interestingly, the first *CCO* gene discovered, formerly known as Vp14 in maize, is connected to the rate-limiting stage of ABA biosynthesis [22]. Likewise, in studies involving *Arabidopsis thaliana*, it was found that five *NCED* genes play essential roles in the initial committed step of ABA synthesis [11]. For instance, *NCED6* and *NCED9* were identified as contributors to ABA synthesis during seed development in Arabidopsis thaliana [23].

The precise roles of CCD genes in cucumber remain unclear, while extensive research on CCO genes has been carried out in Arabidopsis thaliana. However, Arabidopsis is not an ideal candidate for investigating CCD enzymes and their functions because it lacks CCDL proteins. Fortunately, CCDL genes have been identified in *Citrullus lanatus* and *Cucumis melo*, opening up new possibilities for further exploration [24]. Carotenoids are responsible for the vibrant colors in fruits and vegetables, and the CCD enzyme family plays a crucial role in both their production and degradation [25]. With the presence of CCDL genes in *C. lanatus* and *C. melo*, these species are promising candidates for future research on CCD enzymes and their functions.

*Cucumis sativus* L., commonly known as cucumber, is a rich source of vitamins, minerals, nutrients, and bioactive compounds. Beyond its role as a staple food, cucumber has a long history of utilization in traditional and contemporary cultures for its therapeutic properties and applications in beauty and skincare. Originating in Asia, cucumbers are now cultivated in both tropical and subtropical regions. Belonging to the Cucurbitaceae family, which comprises 118 genera and 825 species, cucumbers are highly sought after within this plant family due to their exceptional nutritional value [26].

This research undertook a genome-wide exploration of the *CCO* gene family, examining aspects such as gene structure, domains, motifs, phylogenetic connections, miRNA target locations, and cis-elements. This investigation offers a strong basis for forthcoming studies on the functional roles of *CCO* genes in cucumbers. It's important to note that cucumber has not previously undergone an exhaustive examination of the *CCO* gene family. Therefore, delving deeper into the study of *CCO* genes could yield fresh perspectives for enhancing cucumber's ability to withstand stress.

## Material and method

### Sequences retrieval

The NCBI (https://www.ncbi.nlm.nih.gov) database was used to find the amino acid sequences for the carotenoid cleavage oxygenase (*CCO*) domain. In order to find *CCO* genes, these sequences more specifically the RPE65 domain were used in a BLAST-P (Protein-basic local alignment search tool programme) study using the cucurbita genome database (Version 2) (cucurbitgenomics.org/blast). Twelve sequences all were obtained from the cucumber database using this method. The NCBI CDD (Conserved Domain Database) (http://www.ncbi.nlm.nih.gov/Structure/cdd/wrpsb.cgi) was used with preset settings to validate the retrieved amino acid sequences [14]. The proteins lacking the conserved domain were then carefully analyzed and deleted.

### Determination of physio-chemical properties and subcellular localization of CCO gene

The web programme protparam (https://web.expasy.org/protparam/) was used to find out the length of the carotenoid cleavage oxygenase (*CCO*) proteins as well as their molecular weight isoelectric point (pI), GRAVY value and instability index. From the cucumber genome database, the gene names, chromosomal locations and protein sequences were retrieved. Additionally, the WoLF PSORT programme (https://wolfpsort.hgc.jp/) was used to estimate the subcellular localization of the *CCO* gene [27].

### Gene structure, cis regulatory analysis and motif analysis

The gene structure (Intron–Exon) of the *CCO* genes was displayed using the Gene Structure Display Server (GSDS) (v2.0) (http://gsds.cbi.pku.edu.cn/) by using genomic and CDS sequence from cucurbita genome database [28]. Additionally, the PlantCare database (http://bioinformatics.psb.ugent.be/webtools/plantcare/html) was used to find the cis regulatory elements connected to these genes with upstream 1000 bp promoter region from cucurbita genome database (http://cucurbitgenomics.org/blast) [29]. With a maximum value of 25, motifs were found using the MEME suite programme (http:// meme.sdsc.edu/meme/website/intro.html) and the TBtool was used to display the found motifs.

### Phylogenetic analysis

The Molecular Evolutionary Genetic Analysis (MEGA) software was used to carry out the phylogenetic study. The ClustalW method was used to align the amino acid sequences of *CCO* proteins from *Cucumis sativus*, *Arabidopsis thaliana*, *Cucurbita pepo*, *Cucurbita maxima and Oryza sativa*. These protein sequences were then used to build a phylogenetic tree using the neighbor-joining (NJ) algorithm, with a bootstrapping value of 1000 replications. The generated tree was displayed using the iTOL website (https://itol.embl.de/upload.cgi) [30].

### Evolutionary analysis and chromosomal mapping

Ka/Ks ratios were utilized to calculate the *CCO* genes' divergence time by using TBtool. Default parameters were used, as instructed in the program's instructions. The molecular evolution rate of each gene pair was determined by calculating the ratio of Ka/Ks using paralogous genes. The time of divergence (DT) was calculated by using this T = Ks/2r in which “r” signifies the neutral substiuition rate (6.5*10^–9^).The gene duplication occurrences were examined using MCScanX v1.0 (Multiple Collinearity Scan toolbox) with the default settings. Dual synteny was performed by using two crops i.e. *A. thaliana*, *C. maxima*. The synteny graph was created by using the circus module in TBTool [31]. *CCO's* beginning and ending positions were taken from the Cucumber genome database. Using the TBTool, the chromosomal mapping of the gene was made visible.

### GO ontology analysis

The activities of the *CCO* genes were further validated using GO annotations by gene ontology (GO) term enrichment study. The online programme called ShinyGo v0.741 (http://bioinformatics.sdstate.edu/go/), was used for better understand of *CCO* genes function in cucumber. For visualization of biological, molecular and cellular function, an online database shiny Go was used.

### Transcriptomic analysis

#### Gene expression profiling of phloem content in different parts of cucumber

All *CCO* genes' expression levels were measured in a range of plant organs, including phloem-rich leaves, pedicles, stalks and fruits, as well as under various biological conditions. Using already collected high through put sequencing data, the expression analysis of the *CCO* genes were carried out [32]. NCBI Gene Expression Omnibus (GEO) used for information on the developmental stages and the reactions of the various organs.

### Gene expression profiling of cucumber against cold tolerance

The expression analysis of all *CCO* genes to enhance cold tolerance in cucumber was performed by applying chitosan oligosaccharide through a spray method. This analysis utilized previously gathered high-throughput sequencing data [33].

### Putative miRNA analysis

The cucumber mature miRNA sequence was identified by NCBI geo (https://www.ncbi.nlm.nih.gov/geo/). Using psRNATarget, the CDS sequences of all the *CCO* genes were used to locate the associated micro-RNA (miRNA) sequences. The PsRNA comprehends the function of the CCO genes in cucumber. Shiny Go, an online database was used to visualize biological, molecular, and cellular function.

## Results

### Identification of *CCO* gene in *cucumis sativus*

**Fig. 1:**
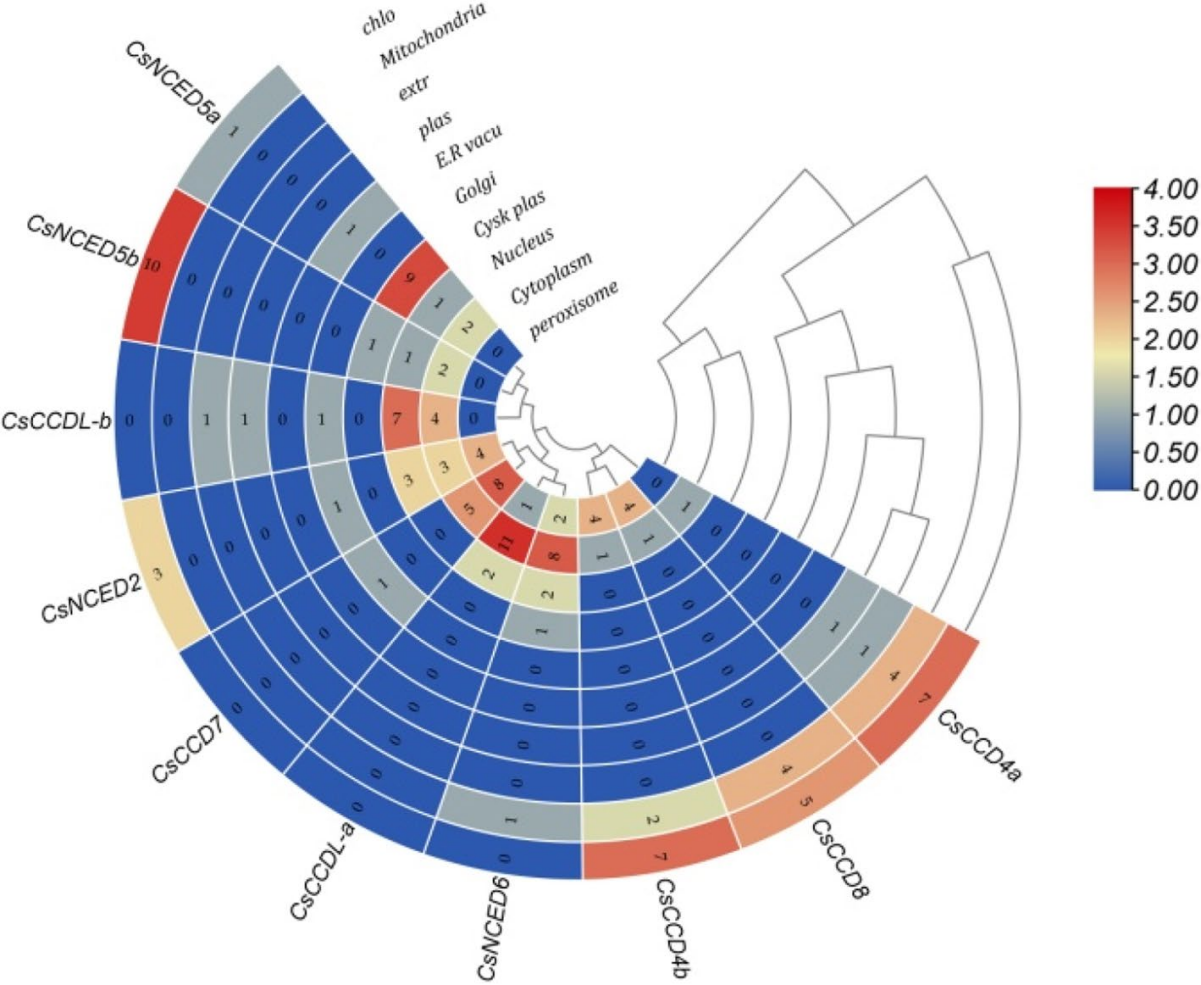
Heat Map illustrating the sub-cellular localization of all *CsCCO* genes to the nucleus, cytoplasm, chloroplast, golgi apparatus, mitochondria, plasmid, peroxisomes of the plant cell. Blue color symbolizes the absence of the relevant gene in the indicated region, grey color predict the minimal functional presence of the relevant gene and red color shows the maximum functional importance of the relevant gene in the indicated region

To identify the *CCO* gene family in *Cucumis sativus*, we conducted a search for these genes within the cucumber genome available in the cucurbit genomics database. Mining of cucumber genome identify 4 *NCED*, 4 *CCD* and 2 *CCDLike* genes in *CCO* gene family. Based on their homologies to *A. thaliana* in phylogenetic tree, these genes were named. The proteins that encoded by identical gene isoforms and with a truncated RPE65-binding domain were eliminated. The pI, MW, GRAVY, instability index and anticipated subcellular localization of these *CsCCO*, as well as other physical and chemical details, are shown in Fig. 1 and Table 1. The protein length of *CsCCO* is in between 91–614 amino acid residues while molecular weight was in range of 10.53 to 67.15KDa. The isoelectric point (pI) values of the identified proteins ranged from 4.72 to 8.78. The GRAVY value shows that all proteis are hydrophilic in nature based on their negative value. In contrast to the remaining proteins, which were determined to be unstable based on the instability index study, *CsCCD4a*, *CsCCD4b*, *CsCCDL-a*, *CsCCD7*, and *CsCCD8* displayed protein stability (as demonstrated by an instability index of less than 40). Predictions of subcellular localization indicated that the majority of *CsCCO* proteins might have a role in the cytoplasm and chloroplasts.

**Table 1:** Non-redundant genes found in the cucumber genome include information on the *NCED*, *CCD*, and *CCDLike* genes in the *CCO* gene family. Abbreviation: AA: Amino acid, Mw: Molecular weight, pI: Isoelectric poin

**Source Accession No**	*CCO* gene Ids	**Chromosome**		**Direct** **Ion**	**Instability index**	Gravy value	**Size (AA)**		**pI**	**Mw**
		**No**	**Location**				**Genome**	**Peptide** **Amino acid**		**(KD)**
**Csa6G523440.1**	CsNCED6	6	28,110,414–28112233	F	42.74	-0.377	1803	600	834	67.15
**Csa1G435760.1**	CsNCED5b	1	16,018,691–16020709	R	43.84	-.0335	1782	593	6.82	66.92
**Csa4G064690.1**	CsNCED5a	4	5,289,846–5,291,369	R	40.24	-0.247	1278	425	5.43	47.77
**Csa2G160620.1**	CsCCD8	2	9,242,639–9,244,360	F	39.98	-0.298	1707	568	8.78	63.02
**Csa7G428120.1**	CsCCD7	7	16,418,892–16,426,079	F	34.00	-0.276	1644	547	5.93	61.33
**Csa4G056640.1**	CsNCED2	4	4,825,005–4827125	R	45.04	-0.236	1776	591	5.83	65.03
**Csa2G373590.1**	CsCCD4a	2	18,727,348–18,731,178	R	37.04	-0.235	1644	547	6.17	60.76
**Csa3G895690.1**	CsCCDL-a	3	38,576,412–38,578,459	R	32.18	-0.425	1110	369	8.68	42.4
**Csa6G106700.1**	CsCCD4b	6	6,987,436–6,992,822	F	37.12	-0.345	1845	614	6.73	69.42
**Csa3G895700.1**	CsCCDL-b	3	38,578,854–38,579,227	F	42.50	-0.677	276	91	4.72	10.53

### Phylogenetic analysis of *CCO* protein

**Fig. 2:**
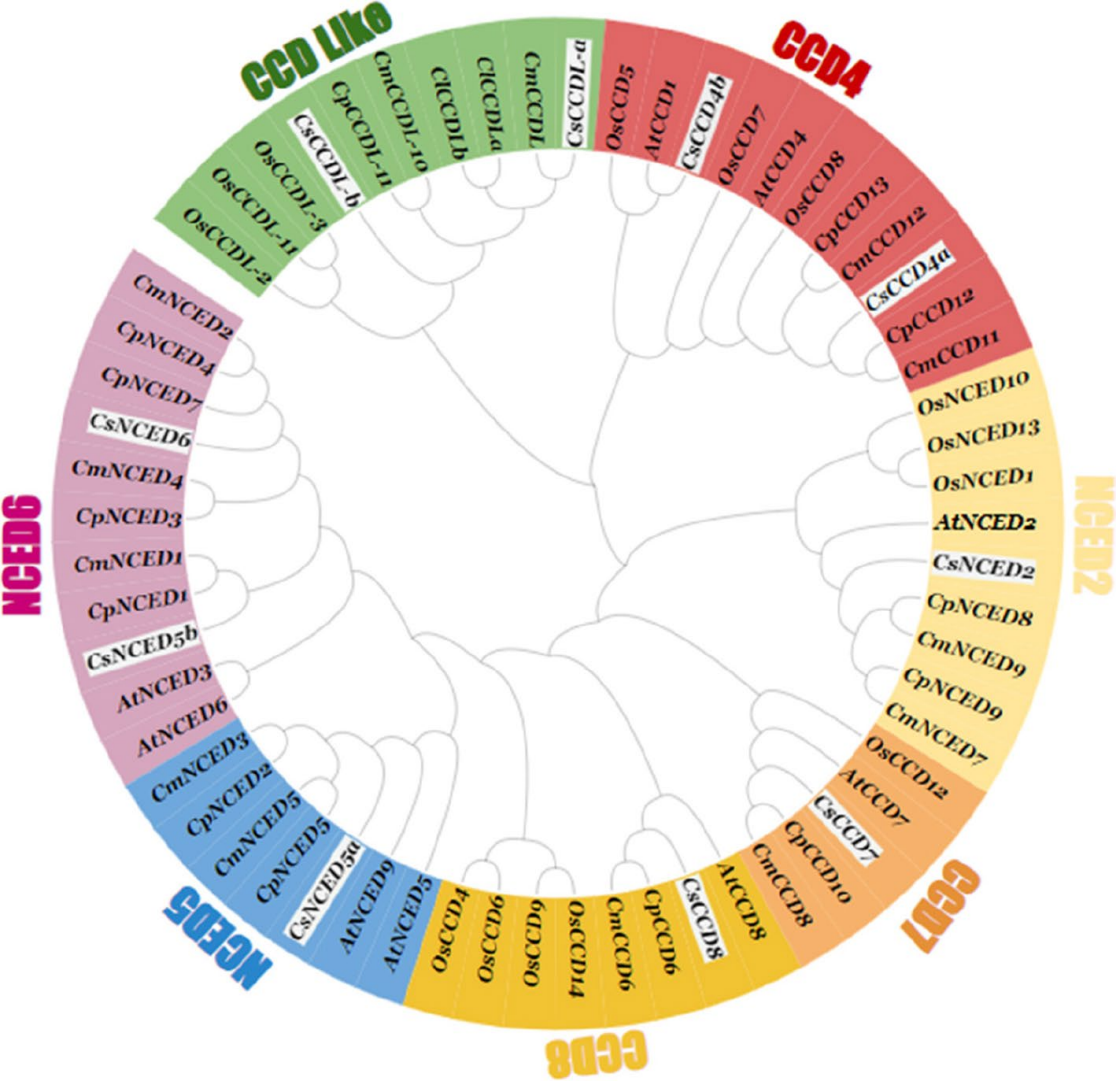
A phylogenetic tree depicting the relationships among *CCO* proteins in four plant species *Cucumis sativus* (Cs), *Arabidopsis thaliana* (At), *Cucurbita pepo* (Cp),*Cucurbita maxima* (Cm) and *Oryza sativa* was constructed using the ClustalW program in MEGA-X. The neighbor-joining (NJ) method was employed for tree construction, with a bootstrap repeat value of 1000 iterations. The phylogenetic tree was visualized using the iTOL online platform. *CCO* proteins from *C. sativus* are highlighted by White Box, while *CCO* proteins from *A. thaliana*, *C. maima*, *C. pepo and Oryza sativa* are depicted in black characters

We explored the evolutionary connections among *CCO* genes in *Cucumis sativus* by utilizing the MegaX software. Phylogenetic tree comprising 61 *CCO* proteins from four distinct species (*A. thaliana*, *C. maxima, C. pepo and oryza sativa*) was constructed using maximum-likelihood (ML) method. This was undertaken to enhance our comprehension of the evolutionary connections among *CCO* proteins. The grouping of phylogenetic tree was based on presence of *Arabidopsis* gene in each clade. The result showed that 61 *CCO* proteins divided into seven subfamilies (*NCED2*, *NCED5*, *NCED6*, *CCD4*, *CCD7*, *CCD8* and *CCDLike*) (Fig. 2). The results demonstrate that the clade *NCED2*, *CCD7*, *CCD8* and *NCED6* each contains one gene of cucumber. In contrast, the clade encompassing *NCED5*, *CCD4*, and *CCDLike* harbors two genes each. *CCDL* genes that were retrieved from another crop, its characteristics were not known yet because these were not present in Arabidopsis. Interstingly, *O.sativa* also containing CCDL genes.

### Gene structure, domain and motif analysis

**Fig. 3:**
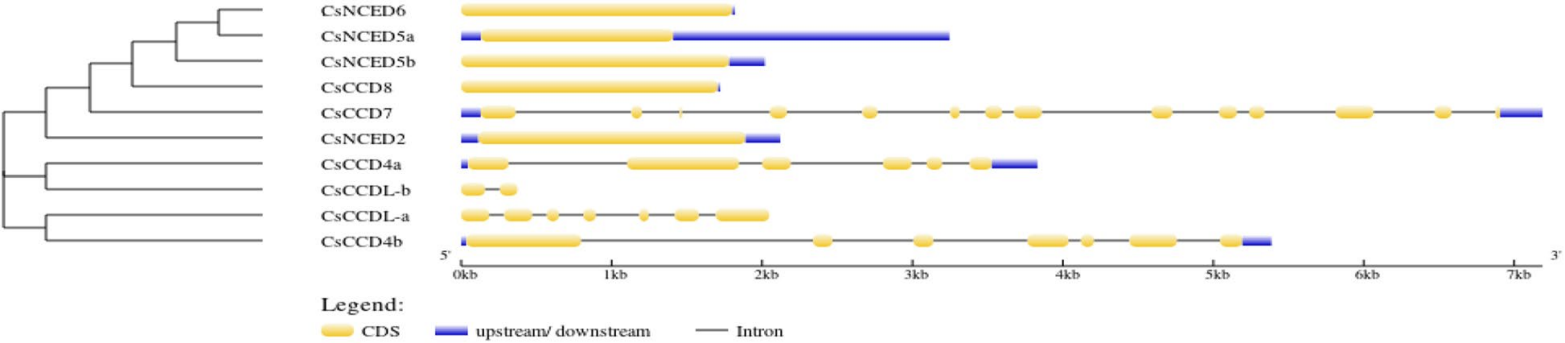
The gene structure of *CCO* genes, revealing the count of introns and exons. Yellow lines indicate the exons, plane line indicate the introns while blue line indicate upstream and downstream

**Fig. 4:**
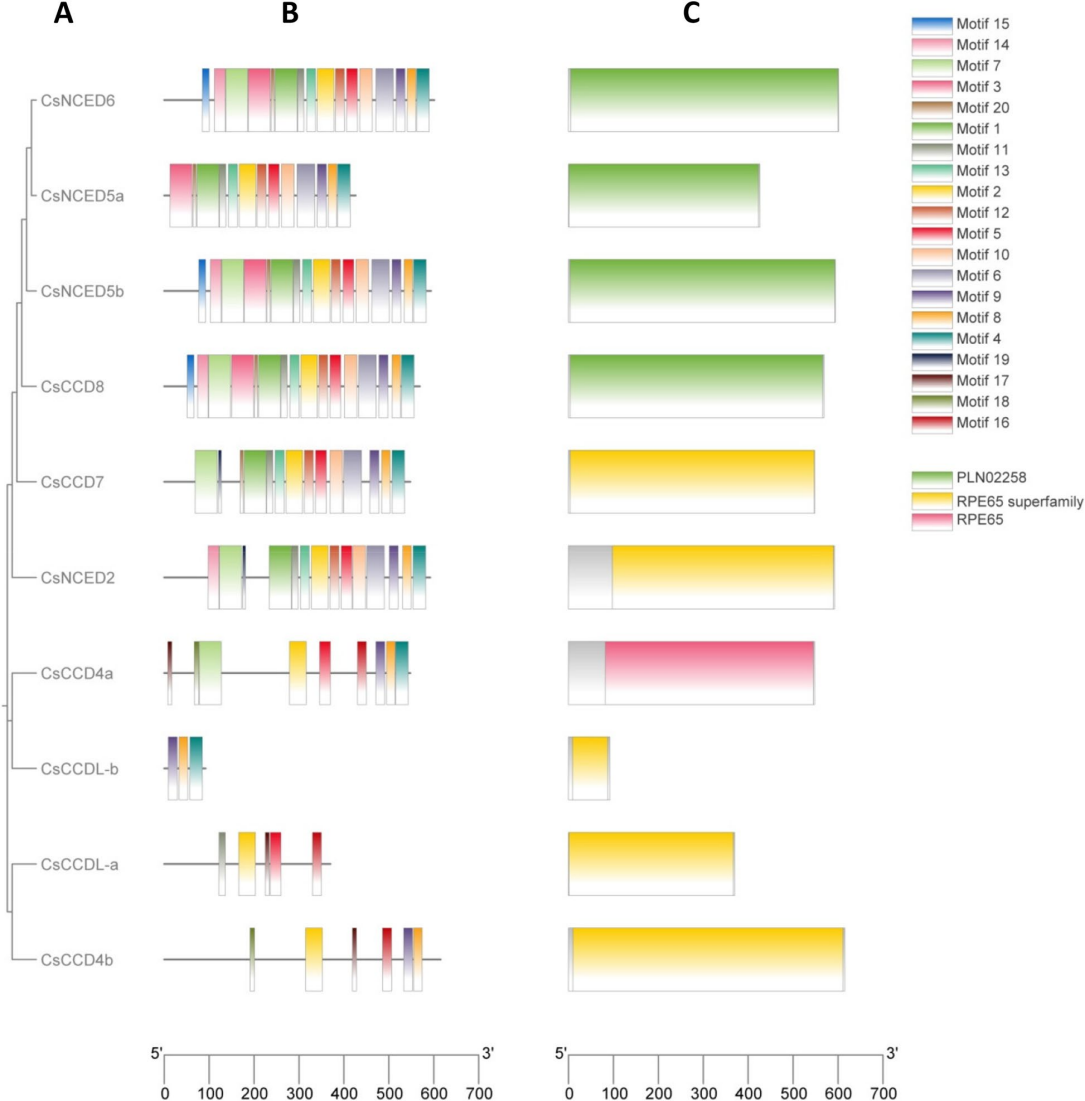
(**A**) A phylogenetic tree was constructed to illustrate the relationships among 10 CCO genes found in Cucumis sativus. **B** To uncover conserved motifs within CCO proteins, an analysis was conducted using the MEME website, and the results were displayed using TBtools. **C** Domain present in CCO proteins was performed was conducted using NCBI conserved database

Analysis of exon–intron patterns and conserved motif composition to gain deeper insights into the gene structure characteristics of the *CCO* gene family, as depicted in Fig. 3. Our investigation of gene structures revealed that the *CsCCO* genes exhibit a range of exon counts, spanning from 1 to 14 exons. It's noteworthy that the *CCD7* subfamily displayed the highest number of both introns and exons. In contrast, the *NCED6*, *NCED2*, and *CCD8* clades were distinguished by their single-exon structure and the absence of introns. Meanwhile, the *CCDlike* displayed 9 exons and 7 introns, while the *CCD4* exhibited a more complex structure with 13 exons and 11 introns as depicted in Fig. 3. Conserved domain analysis exhibited that all of *CsCCO* genes contained a RPE65 domain. The motif analysis of *CsCCO* genes revealed that all members exhibit motifs 2 and 8, with the exception of *CCDL-a*, which lacks motif 8, and *CCDL-b*, which lacks motif 2 in Fig. 4.

### Gene duplication

**Fig. 5:**
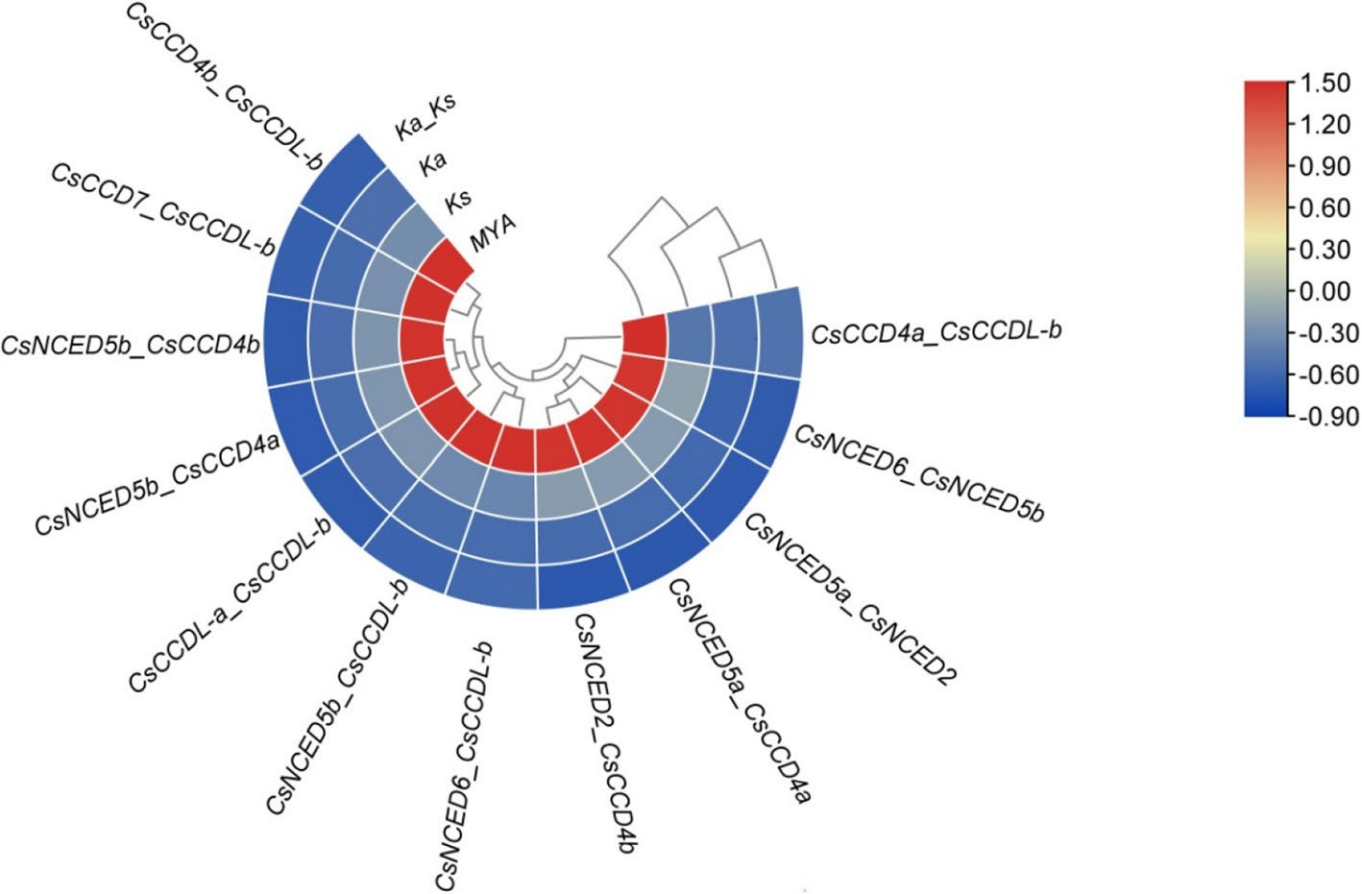
The ratio of mutations involving non-synonymous substitutions (Ka) to mutations involving synonymous substitutions (Ks) is shown as Ka/Ks. On the basis of Ks and Ka values, the gene duplication over selection and evolutionary pressure to paralogous pairings of cucumber *CCO* genes was determined. The red color indicates the duplication in Million years ago while the blue shows ka, ks and ka/ks ratio

The gene duplication of *CCO* was calculated by using TBtool. The Ka/Ks value for gene pairs of *CCO* genes that resulted from both segmental and tandem duplications, and the values were below 1. The ratio of Ka/Ks was range from 0.817998375 in *CsCCD4a_CsCCDL-b* to 0.078261871 in *CsNCED6_CsNCED5b*. This indicates that these genes have evolved under the influence of purifying selection. A possible segmental duplication date was calculated between 266.396 (Million Years ago) for paralogous pair *CsNCED2_CsCCD4b* as highest to 71.50786 (Million Years ago) for paralogous pair *CsNCED6_CsCCDL-b* as lowest in Fig. 5. The MYA value of 71.50786 indicates a recent occurrence of gene divergence. The MYA value of 266.396 indicates a long time ago divergence of these genes from a common ancestors in Fig. 5.

### Synteny analysis and chromosomal mapping

**Fig. 6:**
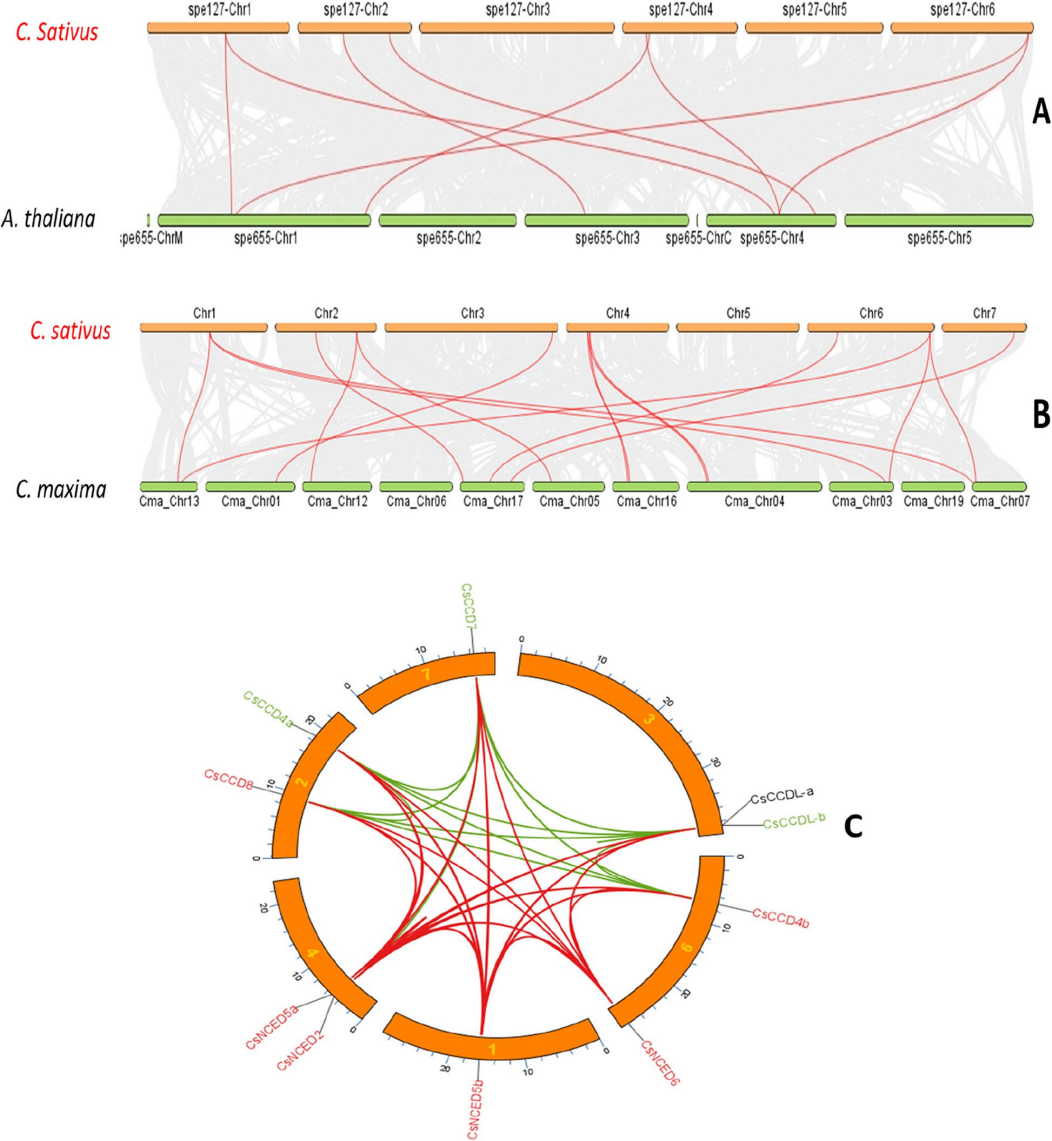
A synteny analysis was conducted on *CCO* genes in cucumber (*Cucumis sativus*) using two plant species as references: *Arabidopsis thaliana* (**A**) and *C. maxima* (**B**). Red and green lines were used to emphasize the syntenic relationships between pairs of *CCO* genes. These hues can be used to clearly depict the degree of similarity between genetic regions. **C** Shows the location of *CsCCO* genes on cucumber chromosomes; lines joining genes on separate chromosomes suggest possible gene duplications

To identify orthologous genes of cucumber in other species, we created a comparative dual synteny of cucumber with Arabidopsis and *Cucurbita maxima*. Through dual synteny analysis of cucumber with Arabidopsis we found 8 orthologue gene pair (Fig. 6A). The total 14 paralouges gene pair have found in cucumber with *cucurbita maxima* (Fig. 6B). The comparison of synteny analysis between *C. maxima* and Arabidopsis revealed a higher number of paralouges genes in *C. maxima*, suggesting a strong evolutionary connection between them. Moreover, an advanced circos plot representing predicted RNAi-related genes in watermelon demonstrated the existence of paralogous genes within the genome (Fig. 6C).

**Fig. 7:**
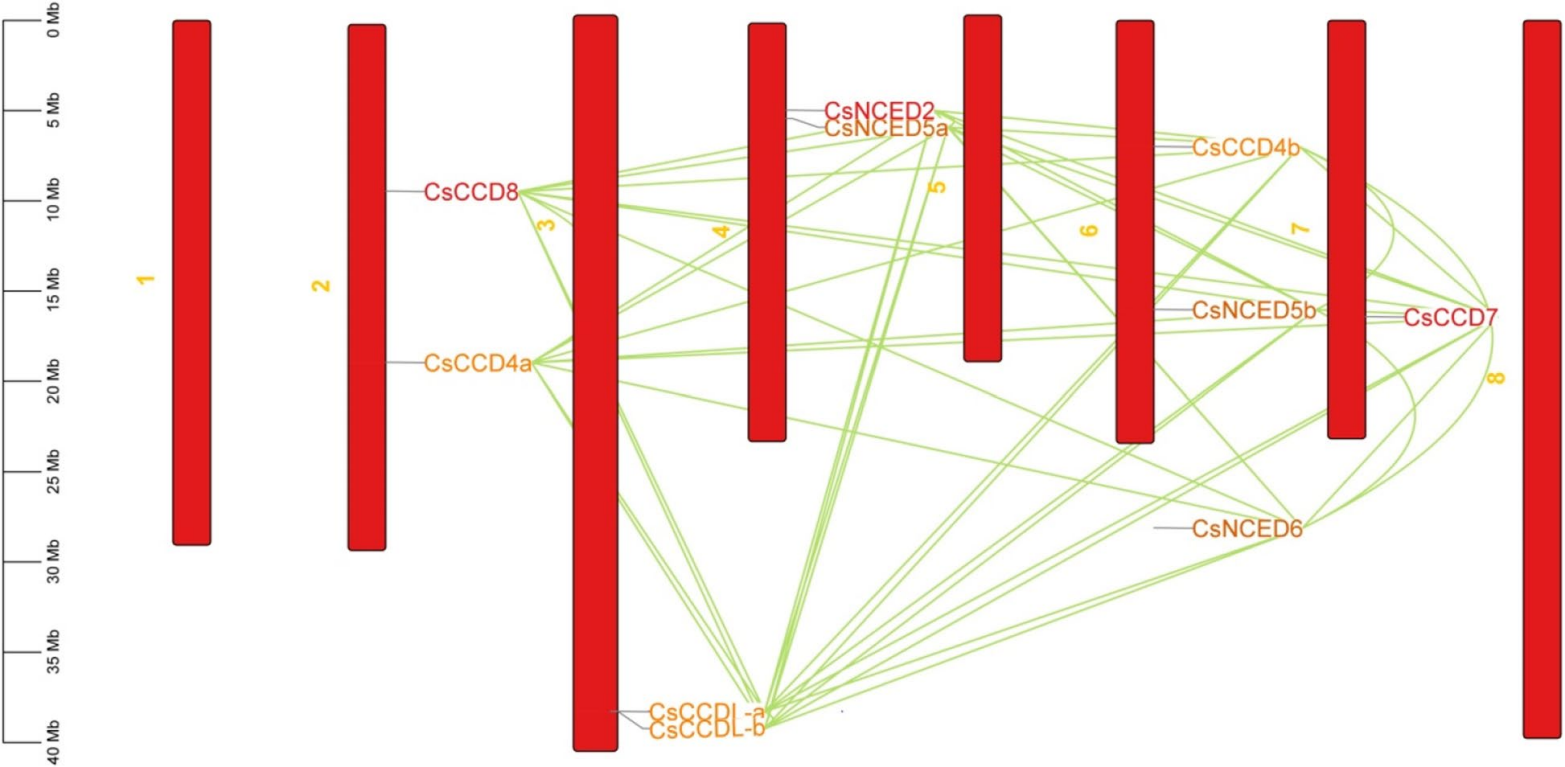
The graphic shows the chromosomal distribution of the cucumber *CsCCO* gene family. Each of the cucumber chromosomes is represented by the red bars in the picture. The spatial organisation and possible interactions between the *CsCCO* genes within the cucumber genome are revealed by this investigation. Conversely, the co-regulation or functional links between the *CsCCO* genes are shown by the green lines. The scale on the map corresponds to the chromosomal distance measured in base pairs (bp). This chromosome map was created using TBtool

Chromosomal distribution indicated that *CsCCO* genes are situated across seven chromosomes, as depicted in Fig. 7. Chromosome 6 (Chr6) harbored three *CsCCO* genes, accounting for 30% of the total. Chromosomes 2, 3, and 4 each contained two *CsCCO* genes, representing 20.0% for each of them. Chromosome 7 hosted a single *CsCCO* gene as Fig. 7.

### Cis element analysis

**Fig. 8:**
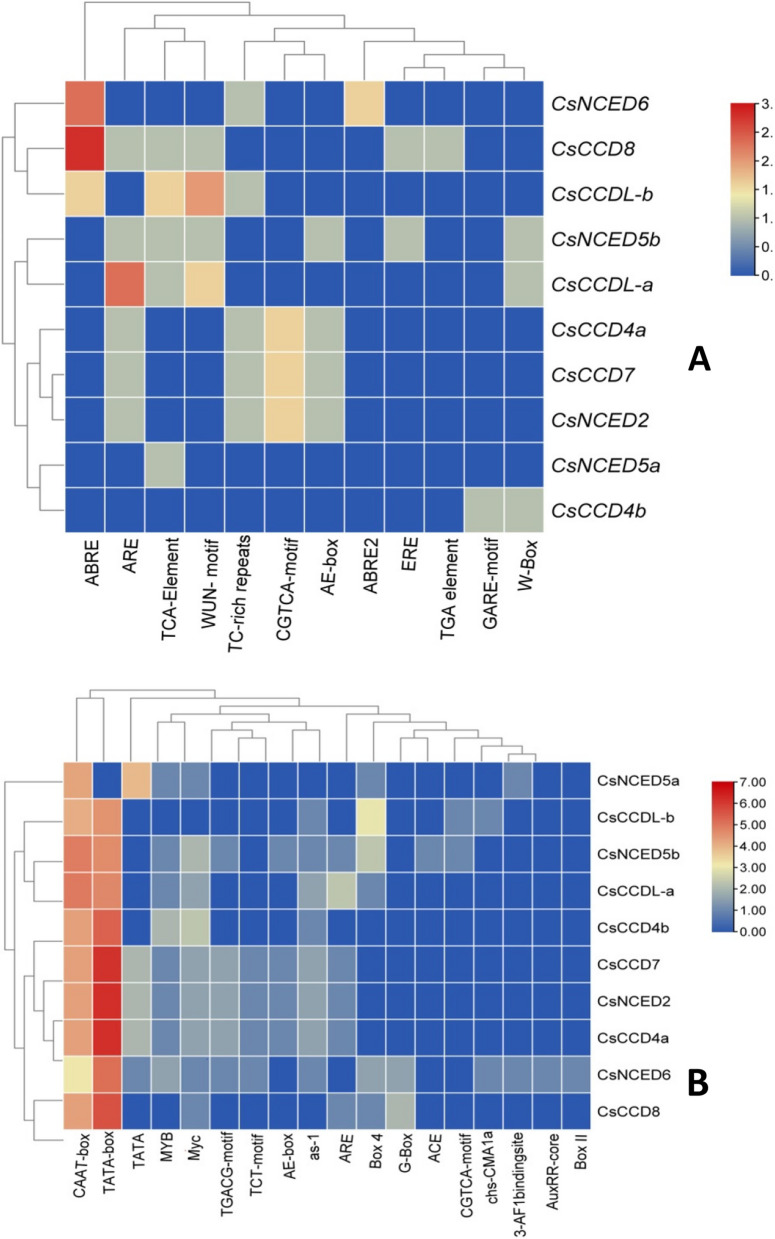
**A** Shows the cis-acting regulatory elements involve in of growth and developmental hormones **B** Involved in gene expression regulation, light response, defense and drought responsive associated elements of 1000 bp promoter sequences of *CCO* genes. The intensity defined by the red (highiest) and light red (lowest) during plant biochemical and physiological processes

Various cis regulatory element were detected in promoter regions of *CCO* genes using PLANT CARE database, these elements plays pivotal roles in gene transcription initiation. Specific cis-regulatory elements associated with growth, development, phytohormone responses and stress induction are illustrated in Fig. 8. Among these elements, The TGACG motif controls genes involved in defense responses and is linked to jasmonic acid signaling. One fundamental promoter element that is necessary for the start of transcription is the TATA-box. While the ARE is an auxin-responsive element that controls genes linked to growth and development, the CAAT-box improves transcription efficiency. Transcription factors for gene regulation are bound by the MYC and MYB motifs; light and stress responses are mediated by G-box; and auxin signaling is linked to growth via AE-box. As-1 is involved in defense mediated by salicylic and jasmonic acids. 3-AF1 binding site controls genes in response to ethylene, while auxin signaling is mediated by AuxRR-core. The roles of Box II, chs-CMA1a, Box 4, and Box 4 may vary, and ACE is involved in the control of genes that react to stress and light. ABA-responsive elements called ABRE and ABRE2 control genes in response to abiotic stress. ERE and ethylene are related, and they affect the ripening and senescence of fruit. In salicylic acid-mediated pathogen defense, the TCA element is essential. Gibberellin-related growth genes are regulated by TGA-element and GARE-motif. Salicylic acid signaling involves W box and WUN-motif, auxin-mediated growth involves AE-box, and different hormonal responses require TC-rich repeats. The CGTCA-motif controls the production of jasmonate acid as a defense mechanism against infections and herbivores. These patterns are essential for the expression of certain genes in plants in response to particular hormonal stimuli as in Fig. 8; Supplementary Table S1.

### MicroRNA target site analysis

All ten genes are targeted by a total of 85 miRNAs. These miRNAs are between 19 and 24 amino acids long. The CsCCD7 is targeted by 14 miRNA (PC-139-3p, csa-miR393b-p3_cme, csa-miR408-5p_ptc, PC-64-5p, csa-miR1510b-3p_gma, PC-10-3p, PC-220-5p, PC-242-5p, PC-34-3p, PC-62-5p, csa-miR167a-p3_cme, csa-miR5816_osa, PC-147-5p, csa-miR172c-p5_cme). Three miRNAs target CsCCDL-b (csa-miR2673a_mtr, csa-miR6483-p3_hbr, and csa-miR6483-p5_hbr). *CsCCDL-a* gene was targeted by 10 mature miRNA, *CsCCD4b* gene was target by 9 mature miRNA. While the other *CCD* gene i.e., *CcCCD4a* and *CsCCD8* were targeted by 9 and 5 mature miRNA respectively. On the other hand, *NCED* genes including *CsNCED2, CsNCED5a, CsNCED5b* and *CsNCED6* were targeted by 10, 6, 6 and 13 mature miRNA as shown in Supplementary Table S2.

### GO annotation

**Fig. 9:**
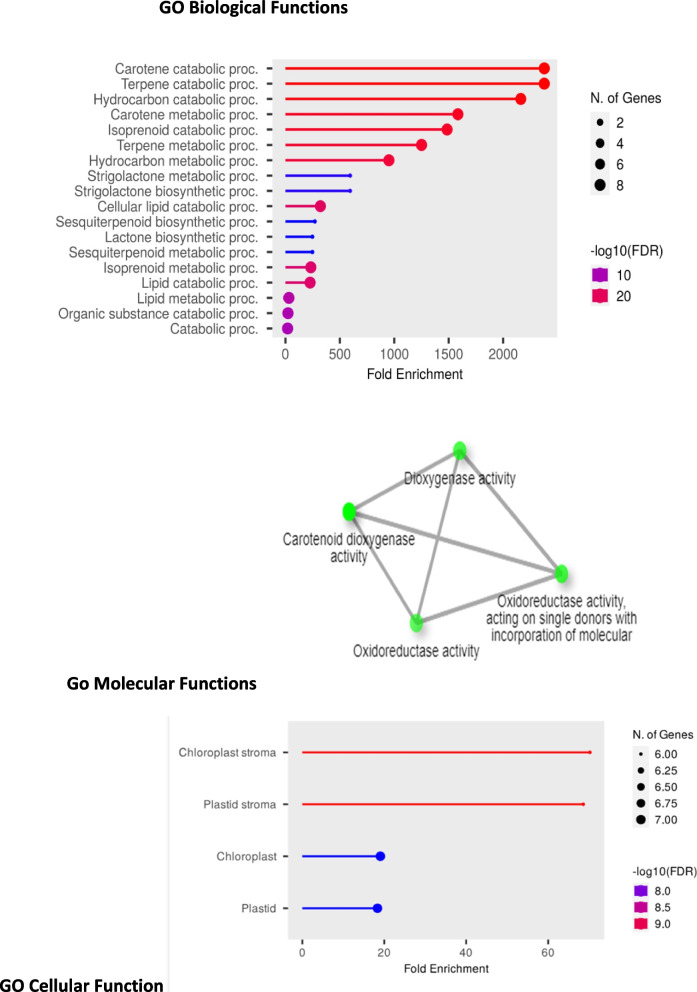
The *CsCCO* genes' overlapping functions are shown by the Fold Enrichment plot and network. This figure illustrates the processes that *CsCCO* genes are mostly involved in and gives a brief summary of their functional distribution

Utilizing GO categories, we elucidated the functions of all CCO proteins, encompassing biological processes, molecular functions, and cellular components. Within the biological process category, a significant majority of protein were implicated in carotene catabolic processes (GO:0016119) and terpene catabolic (GO:0016702). Concerning molecular functions, the enriched terms included oxidoreductase activity (GO:0016702). In terms of cellular components, chloroplast stroma (GO:0009570) and plastids (GO:0005575) was predominantly enriched. These findings underscore the diverse roles that these *CCO* proteins play in cellular metabolism in Fig. 9 and Supplementary Table S3.

### Expression analysis of *CCO* gene for transportation of phloem content in different plant organs in cucumber

**Fig. 10:**
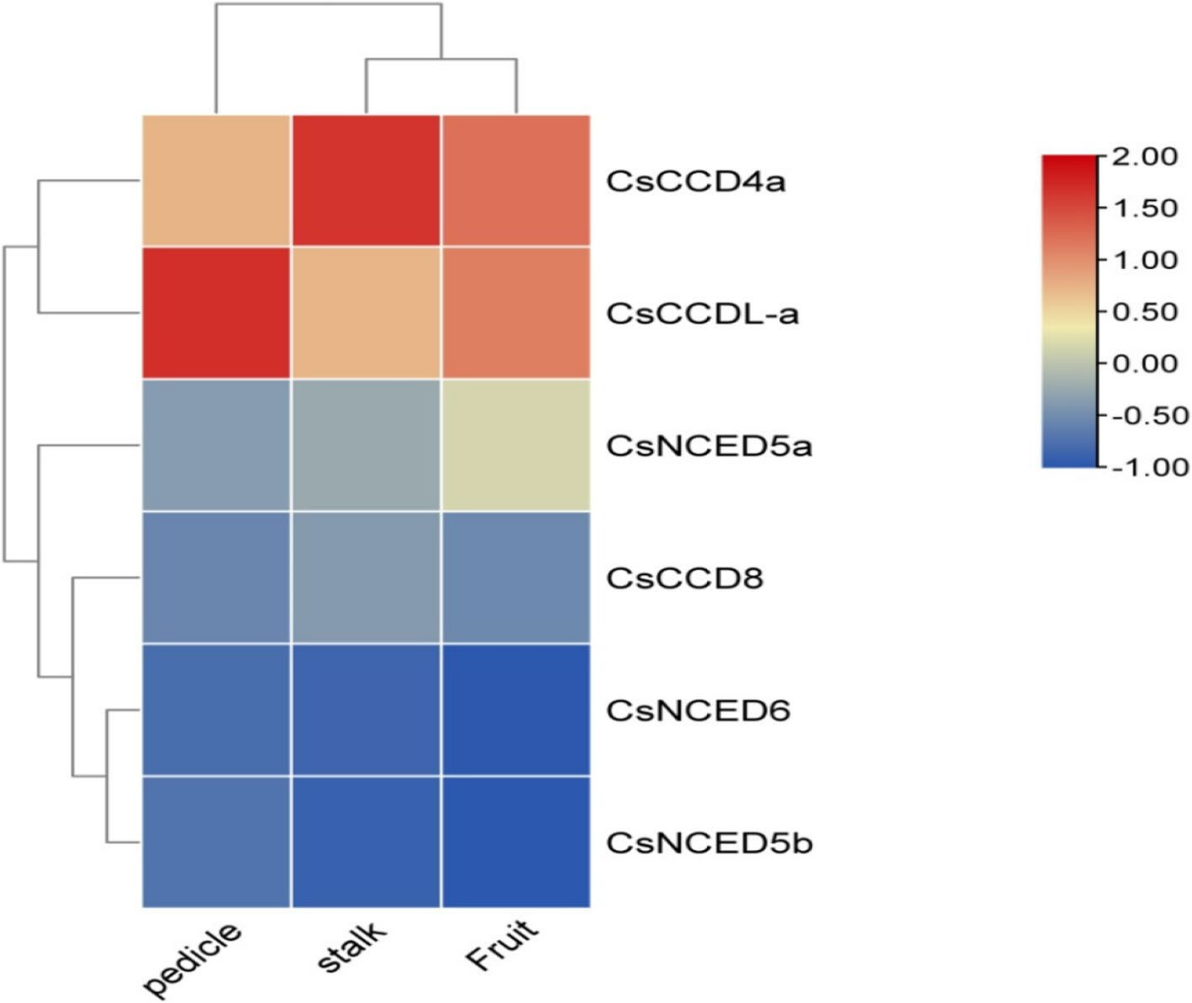
Heatmap showing phloem content in different plant organs. The red color shows the highest gene expression and skin color shows medium expression as well as blue color show lowest gene expression

We utilized previously published transcriptome data of cucumber to create expression profiles for the predicted *CCO* genes in response to phloem content in different organs. Phloem-specific transcript profiling was acquired in three separate organs (pedicle, stalk and fruit) using laser microdissection and RNA-Seq technologies. The data showed the expression of *CsNCED5a*, *CsNCED6, CsNCED5b, CsCCD8* but none of *CsNCED7* and *CsCCDL-b*, *CsCCD4b* and *CsNCED2* genes were expressed. *CsCCD4a* and *CsCCDL-a* have highest gene expression in all three organ fruit, pedicle and stalk as shown in Fig. 10.

### Expression analysis of *CCO* gene showing improved cold tolerance in cucumber by the treatment of chitosan oligosaccharides

**Fig. 11:**
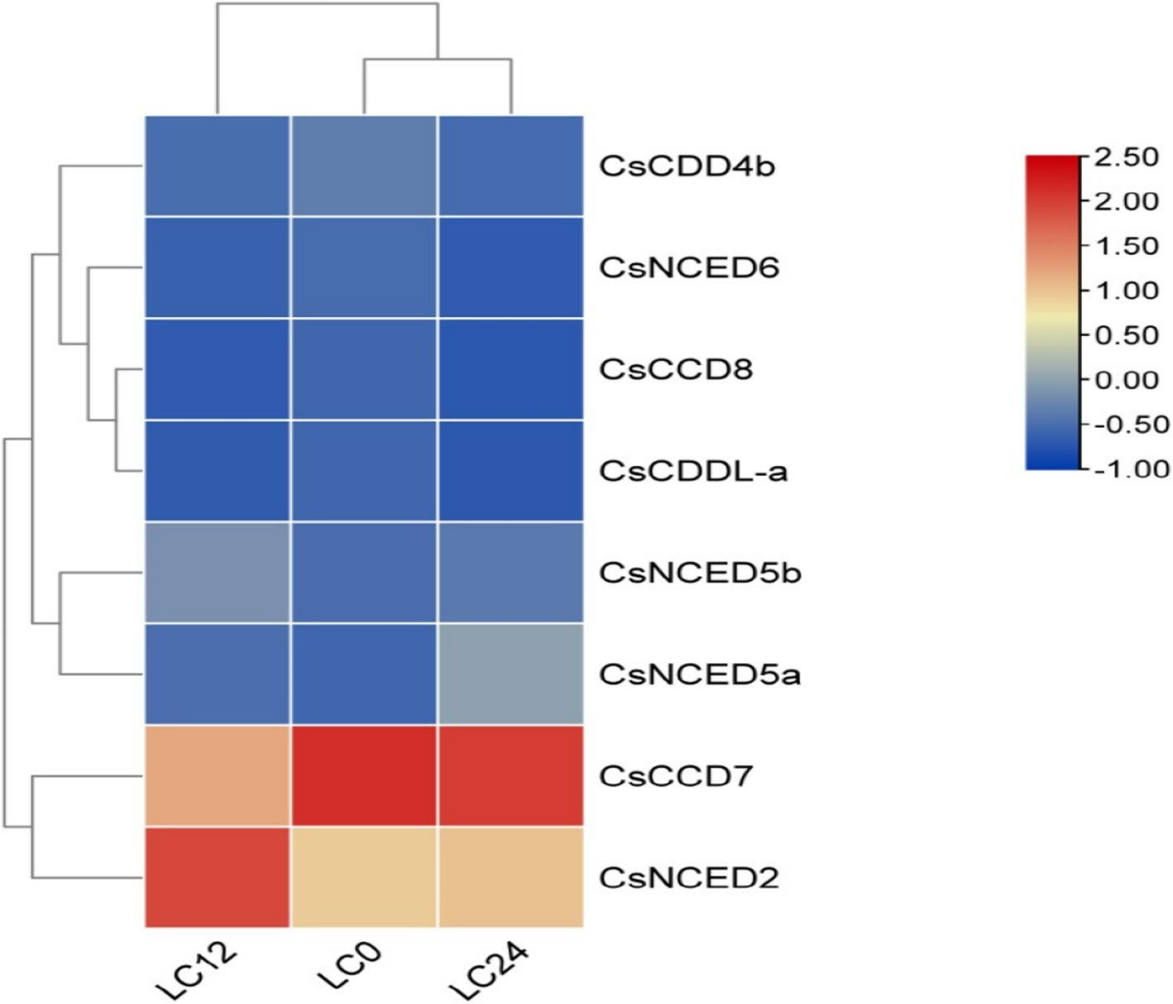
Heatmap showing expression of cucumber seedlings after exposure of chitosan oligosaccharide. The color red signifies highest gene expression, while grey represents a down regulation of gene expression. Blue indicates the null expression. Additionally, the legend provides information about the scale of expression values

To examine the expression patterns of the predicted *CCO* genes in cucumber in response to cold stress, we utilized publicly accessible transcriptome data for our analysis. The data was generated to check the pretreated effect of 50mgl^−1^chitosan oligosaccharide to cucumber seedlings. For transcriptome analysis, samples of seedlings under cold stress were taken at 0, 3, 12 and 24 h. Distilled water served as the control. The data showed expression of *CsNCED6, CsNCED5b, CsNCED5a, CsCCD8, CsCCD7, CsNCED2, CsCCDL-a* and *CsCCD4b* but there is no expression of *CsCCDL-b* and *CsCCD4a. CsCCD7* and *CsNCED2* gene shows highest expression.The maximum expression shows after 3 h of treatment in *CsCCD7* and after 12 h in *CsNCED2* but interestingly also show expression after 0 h of treatment. And rest of the gene *CsNCED5b*, *CsNCED5a, CsNCED6, CsCCD4b* and *CsCCDL-a* were down regulated as shown in Fig. 11.

## Discussion

In the carotenoid metabolic pathway, carotenoid cleavage oxygenase (*CCO*) family can oxidize and split carotenoid molecules for production of apocarotenoids. These apocarotenoids serve as essential component in plant hormones, pigments, aromas, flavors and defensive compounds and hold significant biological importance in the growth and development of plants. Apocarotenoids are linked to hormones such as ABA and SL, as well as non-volatile compounds like crocetin and bixin [34]. The *CCO* gene family is generally present in a wide range of eukaryotic organisms, with a prominent occurrence in a number of plant species. For instance, it has been reported that the *CCO* genes in *Arabidopsis thaliana *[35], *Malus domestica* [15], *Vitis vinifera* [36], *Cucumis melo* [37]. The *CCO* genes comprise a unique class of enzymes that can catalyse the production of smaller molecules from the conjugated double-bond structure seen in carotenoids and apocarotenoids. This procedure has a significant impact on how plants grow, develop, produce high-quality crops, and respond to various environmental challenges. Therefore, it is crucial to learn more about the evolutionary relationships and functional characteristics of these *CCO* genes in plants.

Our investigation involved a comprehensive assessment of the cucumber species' *CCO* gene family, revealing a relatively small set of 10 *CsCCO* genes. Through phylogenetic relationship analysis involving *A. thaliana*, *C. pepo*, *C. maxima* and *Oryza sativa* (as illustrated in Fig. 2), categorized these genes into three primary families: *CCD*, *NCED* and *CCDLike*. These families further consisted of three *CCD* subfamily members (*CsCCD4*, *CsCCD7* and *CsCCD8*) and three *NCED* subfamily members (*CsNCED*5, *CsNCED*2, and *CsNCED*6) and one *CCDLike* which was consistent to previous scholarly efforts within the field [13], This classification framework builds upon earlier research and contributes to a deeper understanding of the *CsCCO* gene family's organization and relationships.

Analysis of physicochemical properties revealed that the majority of *CsCCO* proteins exhibited a length ranging from 91 to 600 amino acids (Table 1), a trend akin to that observed in other plant species [38]. Each *CsCCO* member has unique molecular traits, which are shown in Table 1 after the protein's physicochemical characterization. These differences include things like the GRAVY value, the isoelectric point, and the protein molecular weight. The GRAVY value shows that all proteis are hydrophilic in nature based on their negative value. The RPE65 domain, a distinctive conserved domain within *CCO* proteins, stands as pivotal to the enzymatic oxidation activity associated with carotenoid cleavage [5]. Our examination of conserved domains demonstrated the presence of the RPE65 domain in all *CsCCO* proteins, displaying a parallel distribution pattern within the same subfamily. All *CsCCO* proteins shared a common RPE65 domain, which showed a consistent distribution pattern within the same subfamily, according to the study of conserved domains. A striking similarity in distribution patterns was also discovered within the same subfamily when looking at gene structure and motifs (Fig. 4). No conserved motif was present in the all 10 genes. The motif analysis of *CsCCO* genes revealed that all members exhibit motifs 2 and 8, with the exception of *CCDL-a*, which lacks motif 8, and *CCDL-b*, which lacks motif 2 in Fig. 4. Suggesting their significance as pivotal attributes, these motifs may potentially underlie shared functionalities among them.

Multiple earlier investigations, including those conducted previously, have consistently confirmed the localization of the *CCO* gene on chromosome 7. Analyzing both synteny and dual synteny not only unveiled the interconnectivity of *CCO* genes in various crop species but also highlighted their dispersion across chromosomes 2, 3, 4, 5, 6, 7, and 8. This cumulative research provides a solid groundwork for comprehending the positioning of *CCO* genes and their broader implications across diverse crop [39].

Mainly the *CCO* genes were present in cytoplasm and chloroplast [13]. As, current study also predict that all ten genes of *CCO* were predominantly localized in the cytoplasm as shown in (Fig. 1). Although, seven out of ten genes were also present in nucleus and some genes were also present in other organelles as well. Moreover, a parallel inquiry in *Saccharum* highlighted that specific *CCO* genes exhibited intricate localization patterns, implying diverse roles across distinct organelles [8].

A multitude of insights regarding the evolutionary background and gene structure can be obtained by closely examining the genomes of different species. Furthermore, these analyses facilitate the transfer of genetic information from a taxonomic group with a high degree of research to one with a lower degree of study [40]. The discovery of 14 paralogous genes in CsCCO in this study indicates that genes are replicating via gene duplication. This duplication event provides important new information about gene family expansion, which is frequently seen in the kingdom of plants as a result of tandem and segmental duplications [41].

Gene duplication and differentiation are central mechanisms driving the emergence of novel gene families and functions, with the *CCO* gene family exemplifying this trend across diverse plant species by target specific genes through complementary base pairing with miRNAs, allowing them to regulate gene expression by either degrading the miRNA or inhibiting its translation [42].  Singh A et.al 2023 [42] miRNA plays an important role in biotic [43] and abiotic stress [44]. MicroRNAs (miRNAs) are essential regulatory molecules that are involved in nearly every biological function, including plant development, growth, and responses to biotic and abiotic stress. They have distinct roles and are highly conserved [45]. The study demonstrated that the *CsCCOs* possesses a wide range of miRNAs with diverse functions, such as concentrating on genes implicated in the dysregulation of stress. Plant response to biotic and abiotic stressors is regulated by csa-miR393b-p3_cme [46]. Cucumber yield and size are thought to be regulated by csa-miR393a-p3_cme [46]. It is believed that csa-miR408-5p_ptc regulates plant development, growth, and stress response [47]. During the process of somatic embryogenesis, csa-miR528-5p_ata controls a number of target mRNAs by either encouraging their degradation, inhibiting their translation, or doing both [48]. The findings suggest that miRNAs may have a role in regulating the developmental processes associated with *CsCCO* genes.

Cis-regulatory elements are crucial for regulating transcriptional levels of gene expression and are often found in the promoter region of genes [49]. Studies on cis-regulatory elements showed that motifs like G-box, Box 4, AE-box, and others were present in the largest group, which consisted of 18 elements (58.064%) and have been implicated in metabolism and development. The second-largest category, consisting of 13 elements (41.935%), was associated with stress response and included motifs such as ABRE, MYB, STRE, and TGACG-motif [49].

The GO enrichment analysis of *CsCCO* genes indicates that these genes play important roles in pigment synthesis, vitamin A production, and plant defense mechanisms. It also emphasizes the genes' substantial engagement in carotene metabolic and terpene catabolic processes [50]. Their potential in carotenoid cleavage reactions is further highlighted by their relationship with carotenoid dioxygenase activity. The significant involvement of *CsCCO* genes in many metabolic activities inside the chloroplast stroma, especially those pertaining to photosynthesis and pigment production, is indicated by the high enrichment of these genes in this organelle [51].

Consequently, we analyzed the expression profiles of *CsCCO*s in response to phloem content using quantitative real-time polymerase chain reaction (qRT-PCR) collected fron NCBI geo. Phloem plays a vital role in conveying photosynthetic products and systemic signals between sources and sink organs, crucial for overall plant growth and survival. Cucumber's significance as a model species for studying phloem-related research was emphasized [52]. Notably, *CsCCD4a* and *CsCCDL-a* exhibited notably high gene expression across fruit, pedicle, and stalk organs, potentially contributing to phloem transcript regulation in different plant parts as in Fig. 10. The high gene expression of *CsCCD4a* and *CsCCDL-a* in various plant parts could imply their contribution to regulating phloem-associated transcripts involved in biotic stress defense mechanisms.

Another gene expression from NCBI geo showed the response of *CsCCO* genes under cold stress. Incorporating chitosan oligosaccharide in the study amplified the cold stress response in cucumber seedlings. RNA sequencing data unveiled the expression profiles of *CsCCO* gene under these conditions [33]. Notably, genes such as *CsNCED6, CsNCED5b, CsNCED5a, CsCCD8, CsCCD7, CsNCED2, CsCCDL-a* and *CsCCD4b* exhibited activation or upregulation in response to cold stress and chitosan oligosaccharide treatment. *CsNCED2* and *CsCCD7* showed highest gene expression. This sheds light on their potential roles in the plant's defense and adaptability mechanisms under adverse conditions.

The results of this study offer new perspectives on the functional diversity and evolutionary aspects of the *CCO* gene family in plants. These insights will prove valuable for future research, aiding in the exploration and cloning of these genes. The thorough genome-wide identification and characterization conducted in this study pave the way for additional investigations in this field.

## Conclusion

In conclusion, this comprehensive genome-wide analysis has shed light on the previously unexplored *CCO* gene family in cucumber (*Cucumis sativus* L.). We have identified 10 distinct *CCO* genes, classified into three subfamilies (*NCED*, *CCD* and *CCDLike*) and elucidated their structural, functional, and regulatory properties. The presence of cis-elements associated with growth, development, and stress responses highlights the multifaceted roles of *CCO* genes in cucumber. Moreover, miRNA regulation, purifying selection, and differential expression patterns in response to chitosan oligosaccharides and phloem content provide valuable insights into the functional diversity of *CsCCO* genes. This pioneering study paves the way for future investigations and a deeper understanding of cucumber *CCO* proteins, their roles and potential applications in improving cucumber's response to environmental challenges.

### Supplementary Information

**Additional file 1.**

## Data Availability

All data generated or analysed during this study including Protein sequence with accession numbers (Supplementary data file). Here below are the accession numbers.

## References

[1] Ahrazem O, Diretto G, Argandoña J, Rubio-Moraga Á, Julve JM, Orzáez D, Granell A, Gómez-Gómez L (2017). Evolutionarily distinct carotenoid cleavage dioxygenases are responsible for crocetin production in Buddleja davidii. J Exp Bot.

[2] Kloer D, Schulz G (2006). Structural and biological aspects of carotenoid cleavage. Cell Mol Life Sci.

[3] Schmidt-Dannert C, Umeno D, Arnold FH (2000). Molecular breeding of carotenoid biosynthetic pathways. Nat Biotechnol.

[4] Liang YS, Jeon Y-A, Lim S-H, Kim JK, Lee J-Y, Kim Y-M, Lee Y-H, Ha S-H (2011). Vascular-specific activity of the Arabidopsis carotenoid cleavage dioxygenase 7 gene promoter. Plant Cell Rep.

[5] Ohmiya A (2009). Carotenoid cleavage dioxygenases and their apocarotenoid products in plants. Plant Biotechnol.

[6] Akiyama K, Hayashi H (2008). Plastid-derived strigolactones show the way to roots for symbionts and parasites. New Phytol.

[7] Zhao J, Li J, Zhang J, Chen D, Zhang H, Liu C, Qin G: Genome-wide identification and expression analysis of the carotenoid cleavage oxygenase gene family in five rosaceae species. Plant Molecular Biology Reporter 2021:1–13.

[8] Su W, Zhang C, Feng J, Feng A, You C, Ren Y, Wang D, Sun T, Su Y, Xu L (2021). Genome-wide identification, characterization and expression analysis of the carotenoid cleavage oxygenase (CCO) gene family in Saccharum. Plant Physiol Biochem.

[9] Giuliano G, Al-Babili S, Von Lintig J (2003). Carotenoid oxygenases: cleave it or leave it. Trends Plant Sci.

[10] Marasco EK, Schmidt-Dannert C (2008). Identification of bacterial carotenoid cleavage dioxygenase homologues that cleave the interphenyl α, β double bond of stilbene derivatives via a monooxygenase reaction. ChemBioChem.

[11] Tan BC, Joseph LM, Deng WT, Liu L, Li QB, Cline K, McCarty DR (2003). Molecular characterization of the Arabidopsis 9-cis epoxycarotenoid dioxygenase gene family. Plant J.

[12] Vallabhaneni R, Bradbury LM, Wurtzel ET (2010). The carotenoid dioxygenase gene family in maize, sorghum, and rice. Arch Biochem Biophys.

[13] Wei Y, Wan H, Wu Z, Wang R, Ruan M, Ye Q, Li Z, Zhou G, Yao Z, Yang Y (2016). A comprehensive analysis of carotenoid cleavage dioxygenases genes in Solanum lycopersicum. Plant Mol Biol Report.

[14] Zhang X, Liu H, Guo Q, Zheng C, Li C, Xiang X, Zhao D, Liu J, Luo J, Zhao D (2016). Genome-wide identification, phylogenetic relationships, and expression analysis of the carotenoid cleavage oxygenase gene family in pepper. Genet Mol Res.

[15] Chen H, Zuo X, Shao H, Fan S, Ma J, Zhang D, Zhao C, Yan X, Liu X, Han M (2018). Genome-wide analysis of carotenoid cleavage oxygenase genes and their responses to various phytohormones and abiotic stresses in apple (Malus domestica). Plant Physiol Biochem.

[16] Simkin AJ, Schwartz SH, Auldridge M, Taylor MG, Klee HJ (2004). The tomato carotenoid cleavage dioxygenase 1 genes contribute to the formation of the flavor volatiles β-ionone, pseudoionone, and geranylacetone. Plant J.

[17] Simkin AJ, Underwood BA, Auldridge M, Loucas HM, Shibuya K, Schmelz E, Clark DG, Klee HJ (2004). Circadian regulation of the PhCCD1 carotenoid cleavage dioxygenase controls emission of β-ionone, a fragrance volatile of petunia flowers. Plant Physiol.

[18] Rubio A, Rambla JL, Santaella M, Gomez MD, Orzaez D, Granell A, Gomez-Gomez L (2008). Cytosolic and plastoglobule-targeted carotenoid dioxygenases from Crocus sativus are both involved in β-ionone release. J Biol Chem.

[19] Auldridge ME, McCarty DR, Klee HJ (2006). Plant carotenoid cleavage oxygenases and their apocarotenoid products. Curr Opin Plant Biol.

[20] Raghavendra AS, Gonugunta VK, Christmann A, Grill E (2010). ABA perception and signalling. Trends Plant Sci.

[21] Chernys JT, Zeevaart JA (2000). Characterization of the 9-cis-epoxycarotenoid dioxygenase gene family and the regulation of abscisic acid biosynthesis in avocado. Plant Physiol.

[22] Schwartz SH, Tan BC, Gage DA, Zeevaart JA, McCarty DR (1997). Specific oxidative cleavage of carotenoids by VP14 of maize. Science.

[23] Lefebvre V, North H, Frey A, Sotta B, Seo M, Okamoto M, Nambara E, Marion-Poll A (2006). Functional analysis of Arabidopsis NCED6 and NCED9 genes indicates that ABA synthesized in the endosperm is involved in the induction of seed dormancy. Plant J.

[24] Xue G, Hu L, Zhu L, Chen Y, Qiu C, Fan R, Ma X, Cao Z, Chen J, Shi J (2023). Genome-Wide Identification and Expression Analysis of CCO Gene Family in Liriodendron chinense. Plants.

[25] Kiokias S, Proestos C, Varzakas T (2016). A review of the structure, biosynthesis, absorption of carotenoids-analysis and properties of their common natural extracts. Curr Res Nutr Food Sci J.

[26] Staub JE, Robbins MD, Wehner TC: Cucumber. In: Vegetables I: Asteraceae, Brassicaceae, Chenopodicaceae, and Cucurbitaceae. Springer; 2008: 241–282.

[27] Kavas M, Mostafa K, Seçgin Z, Yerlikaya BA, Yıldırım K, Gökdemir G (2023). Genome-wide analysis of duf221 domain-containing gene family in common bean and identification of its role on abiotic and phytohormone stress response. Genet Resour Crop Evol.

[28] Barre A, Culerrier R, Granier C, Selman L, Peumans WJ, Van Damme EJ, Bienvenu F, Bienvenu J, Rougé P (2009). Mapping of IgE-binding epitopes on the major latex allergen Hev b 2 and the cross-reacting 1, 3β-glucanase fruit allergens as a molecular basis for the latex-fruit syndrome. Mol Immunol.

[29] Bülow L, Hehl R: Bioinformatic identification of conserved cis-sequences in coregulated genes. Plant Synthetic Promoters: Methods and Protocols 2016:233–245. 10.1007/978-1-4939-6396-6_15 27557771

[30] Rehman OU, Uzair M, Chao H, Fiaz S, Khan MR, Chen M (2022). Role of the type-B authentic response regulator gene family in fragrant rice under alkaline salt stress. Physiol Plant.

[31] Mazhar HS, Shafiq M, Ali H, Ashfaq M, Anwar A, Tabassum J, Ali Q, Jilani G, Awais M, Sahu R (2023). Genome-Wide Identification, and In-Silico Expression Analysis of YABBY Gene Family in Response to Biotic and Abiotic Stresses in Potato (Solanum tuberosum). Genes.

[32] Sui X, Nie J, Li X, Scanlon MJ, Zhang C, Zheng Y, Ma S, Shan N, Fei Z, Turgeon R (2018). Transcriptomic and functional analysis of cucumber (Cucumis sativus L.) fruit phloem during early development. Plant J.

[33] Tan C, Li N, Wang Y, Yu X, Yang L, Cao R, Ye X (2023). Integrated physiological and transcriptomic analyses revealed improved cold tolerance in cucumber (Cucumis sativus L.) by exogenous chitosan oligosaccharide. Int J Mol Sci.

[34] González-Verdejo CI, Obrero Á, Román B, Gómez P (2015). Expression profile of carotenoid cleavage dioxygenase genes in summer squash (Cucurbita pepo L.). Plant Foods Hum Nutr.

[35] Iuchi S, Kobayashi M, Taji T, Naramoto M, Seki M, Kato T, Tabata S, Kakubari Y, Yamaguchi-Shinozaki K, Shinozaki K (2001). Regulation of drought tolerance by gene manipulation of 9-cis-epoxycarotenoid dioxygenase, a key enzyme in abscisic acid biosynthesis in Arabidopsis. Plant J.

[36] Lashbrooke JG, Young PR, Dockrall SJ, Vasanth K, Vivier MA (2013). Functional characterisation of three members of the Vitis vinifera L. carotenoid cleavage dioxygenase gene family. BMC plant biology.

[37] Cheng D, Wang Z, Li S, Zhao J, Wei C, Zhang Y (2022). Genome-wide identification of CCD gene family in six Cucurbitaceae species and its expression profiles in melon. Genes.

[38] Zhang S, Guo Y, Zhang Y, Guo J, Li K, Fu W, Jia Z, Li W, Tran LSP, Jia KP (2021). Genome-wide identification, characterization and expression profiles of the CCD gene family in Gossypium species. 3 Biotech.

[39] Yue XQ, Zhang Y, Yang CK, Li JG, Rui X, Ding F, Hu FC, Wang XH, Ma WQ, Zhou KB (2022). Genome-wide identification and expression analysis of carotenoid cleavage oxygenase genes in Litchi (Litchi chinensis Sonn.). BMC Plant Biol.

[40] Auldridge ME, Block A, Vogel JT, Dabney-Smith C, Mila I, Bouzayen M, Magallanes-Lundback M, DellaPenna D, McCarty DR, Klee HJ (2006). Characterization of three members of the Arabidopsis carotenoid cleavage dioxygenase family demonstrates the divergent roles of this multifunctional enzyme family. Plant J.

[41] Fatima S, Cheema K, Shafiq M, Manzoor M, Ali Q, Haider MN, Shahid M (2023). The genome-wide bioinformatics analysis of 1-aminocyclopropane-1-carboxylate synthase (acs), 1-aminocyclopropane-1-carboxylate oxidase (aco) and ethylene overproducer 1 (eto1) gene family of fragariavesca (woodland strawberry). Bull Biol Allied Sci Res.

[42] Singh A, Jain D, Pandey J, Yadav M, Bansal KC, Singh IK (2023). Deciphering the role of miRNA in reprogramming plant responses to drought stress. Crit Rev Biotechnol.

[43] Yin Z, Li Y, Han X, Shen F (2012). Genome-wide profiling of miRNAs and other small non-coding RNAs in the Verticillium dahliae–inoculated cotton roots. PLoS ONE.

[44] Sunkar R, Zhu J-K (2004). Novel and stress-regulated microRNAs and other small RNAs from Arabidopsis. Plant Cell.

[45] Ding Y, Ding L, Xia Y, Wang F, Zhu C (2020). Emerging roles of microRNAs in plant heavy metal tolerance and homeostasis. J Agric Food Chem.

[46] Jiang J, Zhu H, Li N, Batley J, Wang Y (2022). The miR393-target module regulates plant development and responses to biotic and abiotic stresses. Int J Mol Sci.

[47] Gao Y, Feng B, Gao C, Zhang H, Wen F, Tao L, Fu G, Xiong J (2022). The evolution and functional roles of miR408 and its targets in plants. Int J Mol Sci.

[48] Luján-Soto E, Juárez-González VT, Reyes JL, Dinkova TD (2021). MicroRNA Zma-miR528 versatile regulation on target mRNAs during maize somatic embryogenesis. Int J Mol Sci.

[49] Zhou Q, Li Q, Li P, Zhang S, Liu C, Jin J, Cao P, Yang Y (2019). Carotenoid cleavage dioxygenases: identification, expression, and evolutionary analysis of this gene family in tobacco. Int J Mol Sci.

[50] Bao Q, Zhang X, Bao P, Liang C, Guo X, Chu M, Yan P (2021). Using weighted gene co-expression network analysis (WGCNA) to identify the hub genes related to hypoxic adaptation in yak (Bos grunniens). Genes & genomics.

[51] Chengcheng L, Raza SHA, Shengchen Y, Mohammedsaleh ZM, Shater AF, Saleh FM, Alamoudi MO, Aloufi BH, Alshammari AM, Schreurs NM (2022). Bioinformatics role of the WGCNA analysis and co-expression network identifies of prognostic marker in lung cancer. Saudi J Biol Sci.

[52] Zhao J, Li Y, Ding L, Yan S, Liu M, Jiang L, Zhao W, Wang Q, Yan L, Liu R (2016). Phloem transcriptome signatures underpin the physiological differentiation of the pedicel, stalk and fruit of cucumber (Cucumis sativus L.). Plant Cell Physiol.

