# Supplementary material for: Genome-wide identification of CCO gene family in cucumber (Cucumis sativus) and its comparative analysis with A. thaliana
Source: BMC Plant Biol. 2023 Dec 11;23:640. doi: 10.1186/s12870-023-04647-4 (PMC10712067; doi:10.1186/s12870-023-04647-4)
Supplement: Supplementary file 1 — Additional file 1. [file 12870_2023_4647_MOESM1_ESM.docx]

**Supplementary data**

>CsNCED6

MTASPSPLLDHLGFLNSTKSNFPNFNINNLKPITSQNSIIHSALHSPSVLHFPNQTTTSTTTTSTTPNFHKSLSLKHQKSPTAPNWNLLQKTASMALDMVESALVSHELQHPLPKTADPRVQISGNFAPVPEQAVKHYLPVAGTIPDCINGVYLRNGANPLFQPTAGHHLFDGDGMVHAVSINHGSASYACRFTQTQRLVQERRLGRPVFPKAIGELHGHSGIARLLLFYARGVFGLVDHKKGTGVANAGLVYFNDRLLAMSEDDLPYHVRITPSGDLQTVGRYDFDKQLHSTMIAHPKVDPISKELYALSYDVVRKPYLKYFRFSPNGIKSKDVEIPLETPTMMHDFAITENFVVIPDQQVVFKLQEMVKGGSPVIYDKNKKSRFGILPKNATDSKDLIWVESPDTFCFHLWNAWEEPETEEVVVIGSCMTPPDSIFNECEENLKSVLSEIRLNLRTGKSTRRAIIKEEKEQVNLEAGMVNKNRVGRKSRYAYLAIAEPWPKVSGFAKVDLVTGEVKKHIYGGRKFGGEPFFLPKEENSKEEDEGYILAFVHDERTWKSEVQIVNAKDLKVEATIKLPSRVPYGFHGTFVQSNDLQNQA*

>CsNCED5b

MVFSSTSTSPHFVLDQKGLGFPIKPINIVKKKPNQRKEVISSALKVNSPFVFHFPKQPNKGPRITEHYTFKSRHPSHWNLLQKVASTAFDILEQAFLNNHNRNLPKNFDPHFQIVGNYAPVPEQPVSHSLPVTGVIPHWINGVYLRNGANPFFQPLSGHHLFDGDGMIHAVTIGEGRASYACRFTQTERLVQEKSIGRPVFPKAVGELHGHSGIARLLLFYARGLFGLIDHRRGTGVANAGLVYFNGRLLAMSEDDLPYQVRITPSGDLITIGRYNFEGQLNSPMVAHPKIDPDSGELFALSYNFTHKPYLKHFRLTPDGKMSPEIDIPLEIPTMIHDFAITEKFVVIPDQQVVFNLQKMLSGGSPVVYDEKKNPRFGFLPKNATDSSNLTWVDSPADTFCFHLWNAWEETESDDIVVIASCMTPPDSIFNERDEEFKAVLTEIRFNLRTGKSSSRRIISESEHVNLEVGVVNRKRVGRKTQYVYLAITDPWPKACGFAKVDVSSGEVKKYIYGDERYGGEPYFLGREMGFGCEEEDDGHIMVFVHDEKKWRSELQVVNATDLKLEACVELSSRVPYGFHGTFVHANDLIHQA*

>CsNCED5a

MVHAVEFSEAGGVSYACRFTETQRLVQERAYGRPVFPKAIGELHGHSGIARLMLFYARGLFGLVDHNHGIGVANAGLVYFNGRLLAMSEDDLPYQIRVTPAGDLKTVGRFNFDGQLESTMIAHPKLDPVSGEMFALSYDVIQKPYLKYFKFSPEGEKSPDVEIPLPQPTMMHDFAITEKYVVIPDQQVVFKLPEMIRGGSPVVYDKEKTSRFGILDKNATDANAIKWIEAPDCFCFHLWNAWEEPETNEVVVIGSCMTPPDSIFNECEENLKSVLSEIRLNLSTGKSTRRPIITETEQVNLEAGMVNRNLLGRKTQFSYLALAEPWPKVSGFAKVDVLSGEVKKYLYGEQRYGGEPLFLPREGAEAEDDGHILAFVHDEKEWKSELQIVNAMTLELEATVKLPSRVPYGFHGTFISCKDLQKQIR*

>CsCCD8

MQISLQSPPPPLTLCRSSSTIETPPIRKLKVITPPRPPLSLPKKVSPPQRRLNPLQKFAASLLDAVESSLFVDKLPKTIDPAVQISGNFSPVPECSVRHDLEIIGNLPACLRGIYLRNGANPMHAPTGGHHLFDGDGMIHAVTFHSGNKASYSCRFTRTNRLQQEAALGRLVFPKPIGELHGHQGLARLAIFLARAGIGLIDGSKGTGVANAGLVYFNGRLLALSEDDLPYHVQIKDDGDLETIGRFNFNGQINCPMIAHPKVDPISGDLHGLSYNMIKKPYLKYLRFDRFGKKSRDVDITLREPTMIHDFAITENHVVIPDHQVVFKLLEMVRGGSPVVFDPKKTSRFGILPKSGVDEKGIVWIEVPNCFCFHLWNAWEETGGNDEKSIVVVGSCMNPPDSIFNDRDQPLRIELTEIRMDVKSRKVTRRVFGSGMNLEAGQMNRGLVGRKTRFVYMAIADPWPKCSGIAKVDLETGKVKKFLYGEGRYGGEPFYVPENGNWGGDNEKEDGGYIVGFVRDEKRERSEVVVVKAAEMEEVAAVRLPVRVPYGFHGTFVSEEELNGQARN*

>CsCCD7

MADQKQKLNGGLAGRSLVEVTPNPSNGLLSKATDLLEKLFVKLMFDSSKPQHYLSGNFAPVHDETPPITDLPVKGYLPECLNGEFVRVGPNPKFSPVAGYHWFDGDGMIHGLRIKDGKASYVSRYVQTSRLKQEEYFGASKFMKIGDLKGFFGLIMVNMQLLRAKLKVLDVSYGTGTGNTALIYHHGKLLALSEGDKPYVIKVLEDGDLQTLGLLDYDKRLKHTFTAHPKVDPVTGEMFTFGYSHSPPYVTYRVISKDGLMHDPVPITIPAPVMMHDFAITENYAIFMDLPLYFKPKEMVKENKLIFTFDATKKARFGVLPRYAKDDLLIRWFELPNCFIFHNANAWEEEDELVLITCRLENPDLDMVSGSVKEKLENFSNELYEMRFNLKSGLASQRKLSESAVDFPRVNESYTGRKQQYVYGTTLDSIAKVTGIAKFDLHAEPETGKTKIEVGGNVQGLYDLGPGRFGSEAIFVPRVPGTTSEEDDGYLILFVHDENTGKSAVNVVDAKTMSSEPVAVVELPHRVPYGFHAFFVTEEQLQEQGRL*

>CsNCED2

MDSISSPFLSGRNLILSPPISSSLPPISTPIYSVLTEQNVKKNTPPPDADSPSPPLPRPSPPSPPMPRVSSTRRVQPSLPARFFNAFDDLINNFINPPVSPSVDPRYILADNFAPVDELPPTECEVIYGSLPSSLNGAYIRNGPNPQYLPRGPYHLFDGDGMLHSLRISDGRAVLCSRYVKTYKYTLERDAGHPVFPNVFSGFNGLTASAARGAVAVGRILTGQYNPANGIGLANTSLAFFGDRLYALGESDLPYPIRLTPNGDIETLARHDFDGKLTLSMTAHPKVDSDTGEAFAFRYGPLPPFLTYFRFDKNGAKHSDVPILSMNRPSFLHDFAITKKYAVFTDIQIGINPTQMIIEGGSPVGSDPSKISRVGLIPRYANDESKMKWFDVPGLNLIHAINAWDEDDAVVIVAPNILSVEHALERMDLVHALVEKIRIDLKTGIVTRTPLSTRNLDFGVIHPSYVGKKHRFVYAGVGDPMPKISGVVKLEISQEERRDCIVACRIFGPGCYGGEPFFVPRERESSDETEAEEDDGYVVSYVHDENSGESRFIVMDAKSPELEIIAAVKLPRRVPYGFHGLFVKESDLNKL*

>CsCCD4a

MMASMAMGSCIISPFNFKTKHPILPIQPSSKPTLSNFTIRSIASPVHPPSPVVVPLPEIDTSNNHVAWTSIRQDRWEGELSIQGHLPSWLSGTYLRNGPGLWNIGDYNFRHLFDGYATIVKLHFDNGRLIAGHRQIESNAYKAAMKNQKICYREFSEVPKADNFLAYVGELANLFSGASLTDNANTGVVKLGDGRVVCLTETQKGSIMIDPDTLETVGQFEYSDSLGGLIHSAHPIVTDSEFLTLLPDLLNPGYLVVRMEPSSNERKVIGRVNCKGGPAPGWVHSFPVTENYVVVPEMPLRYCAQNLLRAEPTPLYKFEWRPESKAFMHVMCKASGNIVASVEVPLFITFHFINAYEERDEKGRITAVIADCCEHNADPAILDRLRLHNLRDSLKYPLPDARVGRFRIPLDGSGYGKLEAALDPDEHGRGMDMCSFNPAYLGKKYRYAYACGAERPCNFPNTLTKIDLVKKVAKNWYEEGTIPSEPFFVARPGATEEDDGVVISMVSAQNGEGYALLLDGSTFEEIARAKFPYGLPYGLHGCWVPKN*

>CsCCDL-a

MLHALYFKKANNNGKSWEVLYNNRFVETDSFNFEKHEKKRPCFLPTVEGDSVALLFAFLLNWVRFGKFSKDISNTNVFEHSGKLYSIAENHLPHQIDINTLHTLGSWDLNASAWNNRPFTSHPKKAPETGELVMMGVTSTKPFMEVGIISEDGKRMVHKVDVKLGRSCLSHEIGVTKRYNVILDYALTMDFDRLIRGGQEIIVSRFLKYDKKGYTRIGVMPRYGDSDSIKWFNVKPNCCMHLFNCFELNQHEVVVWGCRASDSVLPGPEKGLNKFDWFSQRFNDQKKTVVNEDEDEDNNGGSLLPVAYQWRLNLRTGEVKEKCLIHQSIFMDFPFINLRFTGLPNKFGYAQLLHSSASSNSGIFKHNPS*

>CsCCD4b

MKANSLTSSLPLQFPSPIKLPPPSTGIRFPSSLTPKAISISTPNTHRISINKNDDDDDSIAAFWDYQFLFISQRTETENPAVLRLVDGAIPSDFPSGTYYLTGPGMFSDDHGSTVHPLDGHGYLRAFVFEKDHEVTFMAKYVKTEAKMEEHDPETDRWRFTHRGPFSVLKGGKKLGNTKVMKNVANTSVLRWGGRLLCLWEGGDPYEIRAEDLDTVGKFCAFHGGDDHDSPSRGGGYDGGFWRFAAELLKPVLYGVFKMPPKRLLSHYKVDAQRNRLLVMSCNAEDMLLPTSHFTFYEFDSNFKLLQKKDMVIDDHLMIHDWAFTDNHYILFANRIKLDVIGAMSAISGISPMISALSVNTNKSTSPIYLIPRFGEDSKKDDWKETIVEVPSRLWLLHVGNAFETIHEDGTLDFEIHASSCSYQWFNFKKLFGYNWQTGKLDPSVMNPNETKSKQFPHLVKVSISLSKNGKCEKCSVEPLNQWPKSSDFPVINPKFSGLKHNYLYAATSSGNRRSLPSFPFDMIVKLDTVTNTVRTWFAGNRRFVGEPVFVPKGDKEDDGYLLVVEYAVSIQRCYLIILEAQRFGEADGVVARFEVPKHLNFPIGFHGFWAANT*

>CsCCDL-b

MGMQDEEIKVERHMLEENSYCSGASFVPRENGEEEDDGWIIAHVHNEITNTSQQVYIIDARKFSEEPIAKITLPQRVPYGYHGAFIPHQFE*
